# A Fungal Ascorbate Oxidase with Unexpected Laccase Activity

**DOI:** 10.3390/ijms21165754

**Published:** 2020-08-11

**Authors:** Verena Braunschmid, Sarah Fuerst, Veronika Perz, Sabine Zitzenbacher, Javier Hoyo, Cesar Fernandez-Sanchez, Tzanko Tzanov, Georg Steinkellner, Karl Gruber, Gibson S. Nyanhongo, Doris Ribitsch, Georg M. Guebitz

**Affiliations:** 1 Institute of Environmental Biotechnology, Department of Agrobiotechnology, University of Natural Resources and Life Sciences (BOKU), 3430 Tulln an der Donau, Austria; verena.braunschmid@boku.ac.at (V.B.); veronika.perz@meduni.graz.at (V.P.); sabine.zitzenbacher@perrigo.com (S.Z.); g.nyanhongo@boku.ac.at (G.S.N.); guebitz@boku.ac.at (G.M.G.); 2 Austrian Centre for Industrial Biotechnology (ACIB), 3430 Tulln an der Donau, Austria; sarah.fuerst@boku.ac.at (S.F.); georg.steinkellner@acib.at (G.S.); karl.gruber@uni-graz.at (K.G.); 3 Grup de Biotecnologia Molecular i Industrial, Department d’Enginyeria Química, Universitat Politècnica de Catalunya, Rambla Sant Nebridi, 08222 Terrassa, Spain; javier.hoyo@upc.edu (J.H.); tzanko.tzanov@upc.edu (T.T.); 4 Instituto de Microelectronica de Barcelona (IMB-CNM), CSIC, Campus UAB, 08193 Bellatera, Spain; cesar.fernandez@imb-cnm.csic.es; 5 Innophore GmbH, 8010 Graz, Austria; 6 Structural Biology, Institute of Molecular Bioscience, University of Graz, 8010 Graz, Austria

**Keywords:** ascorbate oxidase, laccase, multi-copper oxidase, ABTS

## Abstract

Ascorbate oxidases are an enzyme group that has not been explored to a large extent. So far, mainly ascorbate oxidases from plants and only a few from fungi have been described. Although ascorbate oxidases belong to the well-studied enzyme family of multi-copper oxidases, their function is still unclear. In this study, *Af*_AO1, an enzyme from the fungus *Aspergillus flavus*, was characterized. Sequence analyses and copper content determination demonstrated *Af*_AO1 to belong to the multi-copper oxidase family. Biochemical characterization and 3D-modeling revealed a similarity to ascorbate oxidases, but also to laccases. *Af*_AO1 had a 10-fold higher affinity to ascorbic acid (*K_M_* = 0.16 ± 0.03 mM) than to ABTS (*K_M_* = 1.89 ± 0.12 mM). Furthermore, the best fitting 3D-model was based on the ascorbate oxidase from *Cucurbita pepo* var. *melopepo*. The laccase-like activity of *Af*_AO1 on ABTS (*V_max_* = 11.56 ± 0.15 µM/min/mg) was, however, not negligible. On the other hand, other typical laccase substrates, such as syringaldezine and guaiacol, were not oxidized by *Af*_AO1. According to the biochemical and structural characterization, *Af*_AO1 was classified as ascorbate oxidase with unusual, laccase-like activity.

## 1. Introduction

Ascorbate oxidases catalyze the oxidation of ascorbic acid to dehydroascorbate (DHA), however, their specific function has not yet been fully elucidated [1,2]. There are indications, that ascorbate oxidases are involved in signaling pathways in plants since DHA induces stomata closure in tobacco [3]. Mostly, the functions of ascorbate oxidases are, however, not expected in the oxidation of ascorbic acid, but rather in the reduction of oxygen levels [2]. Maintenance of a stable redox state requires the control of oxygen levels, especially where oxygen is produced by photosynthesis. Furthermore, in some tissues hypoxia is essential to keep stem cells in their pluripotent state [2]. In fungi, the functions of ascorbate oxidase are even less explored. There are indications that they might be involved in plant pathogenesis [4] or fungal development [5,6]. The biotechnological applications of ascorbate oxidases are consequently limited. The most common application is for biosensors to detect ascorbic acid, an important dietary and physiological antioxidant [7].

Ascorbate oxidases belong to the enzyme family of multi-copper oxidases (MCO). The MCO family is one of the earliest and best-studied enzyme groups [8,9]. As their name suggests, MCOs utilize copper to oxidize various substrates, concomitantly reducing oxygen to water [10]. The MCO family is highly diverse and includes, besides ascorbate oxidases (EC 1.10.3.3), laccases (EC 1.10.3.2), ceruloplasmin, and ferroxidases (EC 1.16.3.1) [1]. The enzyme groups differ mainly in their substrate specificity. The structure of MCOs is largely conserved across the various groups. Most MCOs, like ascorbate oxidases and laccases, consist of three domains, except ceruloplasmin, which is comprised of six domains [10]. Common and mechanistically important features in all MCOs, are the four copper ions organized in two sites. The mononuclear site contains one T1 or blue copper-ion and is the site of substrate oxidation. The redox potential of the T1 copper is crucial for the catalytic efficiency of the enzyme [11]. From the T1 copper, the electrons are transferred via a conserved His-X-His motive to the second copper-containing site, the trinuclear site. The trinuclear site contains one T2 copper and a pair of T3 copper ions. There, a four-electron reduction of dioxygen to water takes place [12,13]. Conserved domains, typical for MCOs, coordinate the copper ions, and facilitate the identification of MCO family members [14].

So far, most ascorbate oxidases were found and studied in plants [2], only a handful were found in fungi [1,4,15] and insects [16]. Consequently, literature on fungal ascorbate oxidases is rare. The best described fungal ascorbate oxidase, ASOM from *Acremonium* sp.–HI-25, was described in 1992 [17]. Ascorbate oxidases were also found in the fungi *Myrothecium verrucaria* [18], *Physarum polycephalum* [19], and *Pleurotus ostreatus* [20], but those are atypical, since they probably do not contain copper ions. Xie et al. describe an ascorbate oxidase-like enzyme in the filamentous fungus *Podospora anserine*, whose gene deletion decreased ascorbate oxidase activity [5], but this enzyme was not isolated and therefore not characterized in depth.

Ascorbate oxidases, in contrast to laccases, have narrow substrate specificity, almost exclusively oxidizing ascorbic acid to DHA, but can also accept other lactone-ring containing substrates [21]. The high substrate specificity is probably due to their rather small substrate cavity. One histidine and two tryptophan residues stabilize the lactone ring of ascorbic acid in the cavity [22,23].

In contrast to ascorbate oxidases, laccases have attracted a lot of attention [1]. Since their discovery in the sap of the Japanese lacquer tree (*Rhus vernicifera*) in 1883 [8], laccases were found not only in plants, but also in fungi, and more recently in bacteria and insects [12,24]. The wide range of potential laccase-substrates in nature is reflected in the high diversity of industrial applications, ranging from textile and food processing to chemical synthesis [9].

Ramos et al. identified *Aspergilli* fungi as a good source for the identification of novel MCOs after thorough genomic analysis [25]. Takeda et al. expressed an ascorbate oxidase (ASOM) in *Aspergillus nidulans* [26]. Furthermore, two protein sequences of *A. nidulans* (or *Emericella nidulans*) have been identified as ascorbate oxidase [1], but those have not been characterized in depth. Furthermore, *Aspergilli* have been used to express laccases from other fungi [27]. A few laccases native to *Aspergilli* have been described as well [28,29,30]. For *A. flavus*, for example, five putative laccases have been identified by genome sequencing [31]. Some laccases from *A. flavus* have already been characterized and applied, but lack of protein sequences impedes the classification of these enzymes [32,33,34,35].

This paper focuses on the characterization of an oxidoreductase from *Aspergillus flavus* to elucidate its classification within the MCO family. We found that *Af_*AO1 was highly active on ascorbic acid, but also oxidized 2,2′-azino-bis(3-ethylbenzthiazoline-6-sulfonic acid) (ABTS), one of the most commonly used laccase substrates. A structural modeling of *Af_*AO1 suggested a high similarity to ascorbate oxidase of plants. Therefore, we concluded that *Af_*AO1 is a putative ascorbate oxidase with unprecedented laccase-like activity.

## 2. Results

### 2.1. Sequence Analyses and Copper Content Determination

In the genome sequence of the saprotrophic and pathogenic fungus *Aspergillus flavus*, five putative laccases were found (Ref. seq.: XP_002384796.1, XP_002382290.1, XP_002382290.1, XP_002381510.1, XP_002378028.1) [31]. Of the five putative laccases found in *A. flavus* four contain the signal for extracellular expression. Out of these laccases *Af*_AO1 was best expressed in *Pichia pastoris*. *Af*_AO1 has the locus tag AFLA_123160 and was annotated as putative laccase (Ref. Seq.: XP_002381510.1).

**Figure 1:**
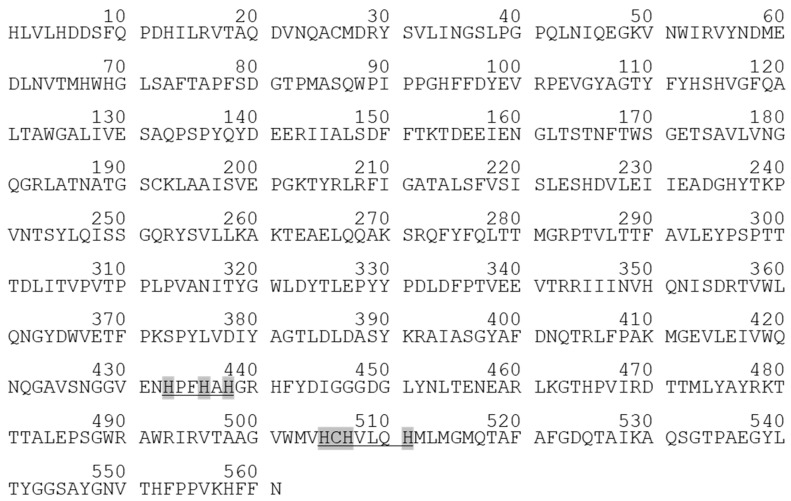
Sequence of *Af*_AO1: conserved regions are underlined, and copper-coordinating residues marked in grey. The sequence is displayed, as it was expressed without signal peptide.

*Af_*AO1 contained 3.3 mol copper per mol of enzyme, which is characteristic for MCOs [1,11,36]. Furthermore, the protein sequence of *Af_*AO1 comprised the typical MCO consensus sequences essential for the binding of the four copper ions (Figure 1) [14] and an absorbance spectrum typical for laccases (Figure S1). Therefore, *Af_*AO1 was identified as a member of the MCO enzyme family. Additionally, *Af_*AO1 contained a conserved region typical for the ascorbate oxidase fungal superfamily [37].

### 2.2. Redox Potential and Substrate Spectrum

**Figure 2:**
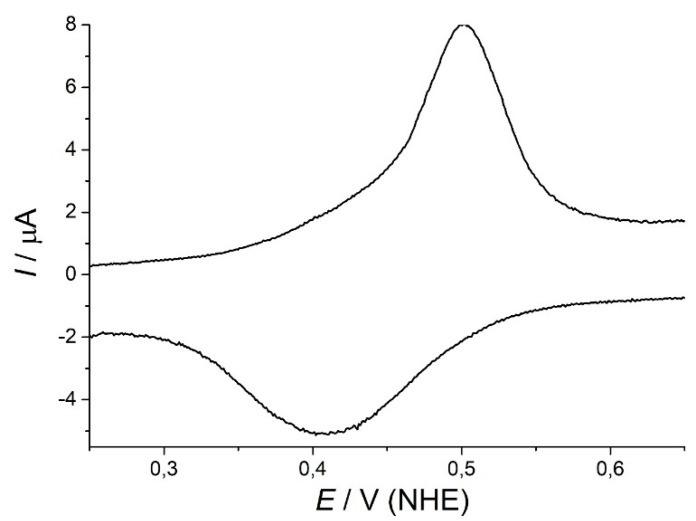
Redox potential of *Af*_AO1: Cyclic voltammogram of *Af*_AO1 at 10 mV·s^−1^, exhibiting an oxidation peak at 500 mV and a reduction peak at 410 mV, indicating a redox potential at 445 ± 10 mV against normal hydrogen electrode (NHE).

The redox potential of an MCO is a characteristic parameter for these enzymes and an important factor for its substrate range [11]. Cyclic voltammetry (CV) was performed to elucidate the redox potential of *Af*_AO1 (Figure 2). The CV of *Af*_AO1 showed an oxidation peak at 500 and a reduction peak at 410 mV indicating a redox potential (E_0_) of 455 ± 10 mV against normal hydrogen electrode (NHE) for the T1 copper ion of *Af_*AO1.

**Figure 3:**
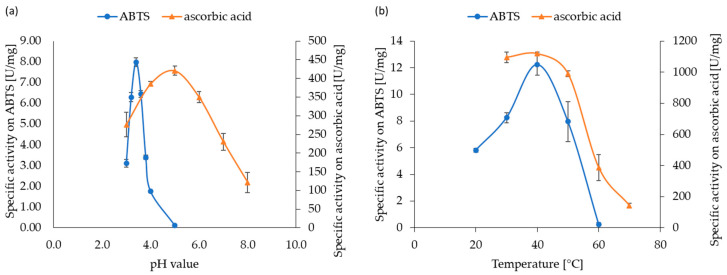
Optimum reaction conditions of *Af*_AO1: (**a**) pH optimum of *Af*_AO1 on ABTS was determined at 25 °C and that on ascorbic acid at 30 °C. (**b**) Temperature optimum of *Af*_AO1 on ABTS and ascorbic acid was measured at the respective optimal pH values of pH 3.4 and pH 5, respectively. Experiments were conducted in triplicates. The error bars depict standard deviation.

A screening of various typical MCO-substrates showed a high specificity of *Af*_AO1 for ascorbic acid and the typical laccase substrate, ABTS. Interestingly, *Af*_AO1 did not oxidize any other tested laccase substrates (Table 1 and Figure S2). Accordingly, the activity of *Af*_AO1 was further characterized on ABTS and ascorbic acid (Figure 3).

### 2.3. Enzyme Optima and Kinetics

On ABTS, *Af*_AO1 showed the highest activity at a pH-value of 3.4, while it preferred a pH of 5.0 for the conversion of ascorbic acid. The temperature optimum at 40 °C, on the other hand, was identical for both substrates (Figure 3). Results for enzyme kinetics differed strongly for the conversion of ABTS and ascorbic acid. Kinetic analyses were performed at the optimal pH value for each substrate. The maximum velocity *V_max_* of *Af*_AO1 on ABTS was 11.56 ± 0.15 µM/min/mg and the affinity of the enzyme to ABTS was moderate, with a *K_M_* of 1.89 ± 0.12 mM. On ascorbic acid, *Af*_AO1 exhibited a significantly higher *V_max_* of 424.89 ± 23.19 µM/min/mg. The affinity of *Af*_AO1 to ascorbic acid was approximately ten times stronger than to ABTS, with a *K_M_* of 0.16 ± 0.03 mM (Figure S3). *Af*_AO1 retained 70% of its ascorbate oxidase activity after 24 h at 40 °C (Figure S4).

### 2.4. Modeling of Af_AO1

**Figure 4:**
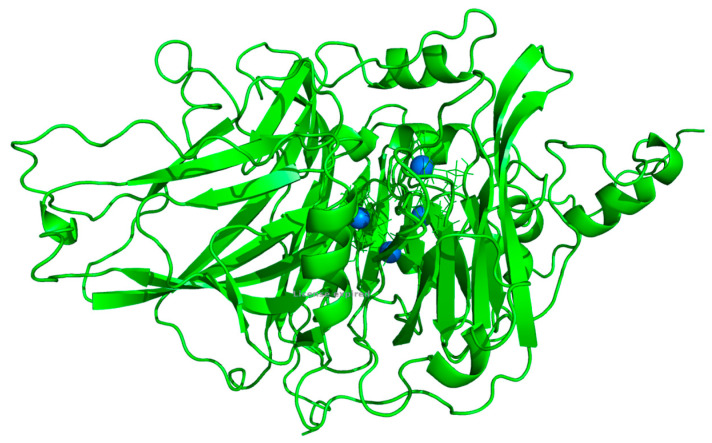
Model of *Af*_AO1: Proposed 3D-structure of *Af*_AO1 based on the structure of the ascorbate oxidase from *Cucurbita pepo* var. *melopepo* (PDB Code: 1ASQ [42]). Copper atoms are displayed in blue and coordinating residues are represented as sticks.

For structural investigation of *Af_*AO1, we modeled its 3D-structure based on known homologous structures derived from different alignments. Despite the overall moderate sequence identity (~32%) and similarity (~50%) to available template structures, we were able to generate a model of *Af*_AO1. The five models built on two laccases from *Steccherinum murashkinskyi* (5E9N [38] and 5MEW [39]), a laccase from *Lentinus tigrinus* (2QT6 [40]), a laccase from *Cerrena maxima* (3DIV [41]) and an ascorbate oxidase from *Cucurbita pepo* var. *melopepo* (1ASQ [42]) ranked similarly high according to their Z-score for dihedrals and overall packing. Manual inspection of the quality of the models regarding overall alignment and the position of the histidines and copper ions, revealed that the best model was built on the structure of the ascorbate oxidase from *Cucurbita pepo* var. *melopepo* (PDB Code: 1ASQ [42]) (Figure 4). This model was used as a representative structure and for model analysis. Of 561 target residues 453 (80.7%) were aligned to the template residues. Among these aligned residues, the sequence identity was 32% and the sequence similarity was 47% (Figure S5). In this model, the copper ions were in a similar position compared to the ones in the template, which was not always the case at different modeling attempts using alternate alignments and selected template structures. Furthermore, the overall amino acid placements, regarding the copper-binding sites, were similar to the template structure in the model. The most differences compared to the template were near the proposed active site (Figure S6).

## 3. Discussion

In this study, we investigated a novel ascorbate oxidase *Af_*AO1 from *A. flavus*. The gene sequence was published as part of a genome sequencing study by Nierman et al. [31]. MCO members contain four copper ions that are coordinated by conserved amino acid residues. These conserved regions make it relatively easy to identify an enzyme as belonging to the MCO family [14]. The *Af_*AO1 sequence contained all MCO-characteristic copper-coordinating sequences. Furthermore, analysis of copper content showed that the expressed enzyme contained 3.3 mol copper per mol enzyme. This is consistent with the copper content expected for MCOs [1]. Therefore, *Af_*AO1 was identified as a member of the MCO family.

The determination of function from sequence is considerably more difficult for MCO members than the grouping into this family [14]. *Af_*AO1 contained a region typical for the fungal ascorbate oxidase family. The fungal ascorbate oxidase superfamily comprises, besides fungal ascorbate oxidases, also plant ascorbate oxidases and laccases, as well as, laccase-like enzymes [37].

At the T1 copper site, MCOs oxidize their substrates, then transfer the electrons to the trinuclear-copper center, where oxygen is reduced to water. The redox potential of the T1 copper site is characteristic for each enzyme and has an impact on its substrate range [11]. The T1 copper site of *Af_*AO1 exhibited a redox potential of 445 ± 10 mV against NHE. Laccases are grouped according to their redox potential. High redox potential laccases have a redox potential above 710 mV. Laccases with a redox potential between 460 and 710 mV are called medium redox potential laccases and all below that are called low redox potential laccases [12]. Fungal laccases are generally found in the medium to high redox potential range, while low redox laccases are mainly bacterial [43]. The differences in the redox potential of laccases can be explained by the big variety of functions, and thereby the wide range of substrates, of laccases. Ascorbate oxidases generally exhibit a considerably lower redox potential. They mainly oxidize one substrate, ascorbic acid, but do so under different conditions. Therefore, the range of redox potential could be smaller than for laccases. For comparison, the fungal ascorbate oxidase ASOM (from *Acremonium* sp.) has a redox potential of 197 mV [44] and the ascorbate oxidase from zucchini of 139 mV [45]. Cucumber ascorbate oxidase has a relatively high redox potential of 350 mV [46]. According to its redox potential *Af_*AO1 could be either a low redox potential laccase or a high redox potential ascorbate oxidase.

The redox potential usually correlates with the substrate range of an enzyme. Generally, a substance can be oxidized by a substance with higher redox potential. Therefore, the higher the redox potential of an MCO the higher the redox potential of their substrates can be and therefore the range of oxidizable substrates increases. Oxidoreductases are, however, not only restricted by their redox potential, but also by the accessibility of their substrate-binding site [11]. Laccases normally have a very wide substrate spectrum. Ascorbate oxidases, in contrast, are very specific for ascorbic acid and lactone ring containing substrates. This narrow substrate range can be explained by the comparatively low redox potential and by amino acids restricting the access to the active site. The active site of ascorbate oxidases is generally very narrow, and the lactone ring of the substrate is stabilized by a histidine and two tryptophan residues [23]. *Af_*AO1 showed a very narrow substrate range for ascorbic acid and for ABTS. The narrow substrate range is in accordance with its rather low redox potential, compared to that of laccases. On the one hand, the narrow substrate range is unusual for laccases and typical for ascorbate oxidases, suggesting that *Af_*AO1 is an ascorbate oxidase. On the other hand, ABTS is a characteristic laccase substrate and typically not oxidized by ascorbate oxidases, as it does not contain a lactone ring. Xie et al. found that the deletion of the gene of a putative ascorbate oxidase in *P. anserine* reduced its ability to oxidize ABTS and ascorbic acid. This enzyme was accordingly classified as ascorbate oxidase, but with laccase-like activity [5]. To our knowledge, no other ascorbate oxidases with ABTS oxidizing ability have been reported so far.

The pH optimum of laccases depends strongly on the substrate [47]. For the oxidation of ABTS low pH optima of around 3 to 5 are usually reported [48,49,50]. For ascorbate oxidases in plant optimal pH of 5 to 6.5 were reported [51,52,53]. ASOM, the only fungal copper-containing ascorbate oxidase described until now, has a pH optimum of pH 4 or 4.5 on ascorbic acid [15,17]. The pH optimum of *Af_*AO1 on ABTS of pH 3.4 fits very well to reported data for laccases. *Af_*AO1 showed the maximal activity on ascorbic acid at pH 5. This pH value lies within the range expected for ascorbate oxidases, although it is higher than that of ASOM [15,17].

The temperature optimum for *Af_*AO1 on both substrates was 40 °C. For most enzymes, the temperature optimum is not dependent on the substrate. The temperature optimum of *Af_*AO1 was lower than the average temperature optimum of laccases, which is between 50 °C and 70 °C [47]. On the other hand, some laccases with considerably lower temperature optima have been reported as well. A laccase isolated from *Polyporus* sp. for example, exhibited the highest activity at a temperature of 25 °C [54]. For ascorbate oxidases temperature optima range from 37 °C in orange peel [53] to 50 °C in wheat [55]. The fungal ascorbate oxidase ASOM exhibits optimal activity at a temperature of 45 °C [17]. Laccases, because of their high variety in substrates and functions, have a wide variety of environments and conditions that they need to be able to function in. This is reflected in the wide range of optimal temperatures and pH-values. The optimal temperature and pH range of ascorbate oxidases is narrower. Most of the described ascorbate oxidases act inside plants and the environment for their functionality is thereby restricted and only vary slightly from plant to plant. The temperature optimum of *Af_*AO1 was therefore in the range of both laccases and ascorbate oxidases.

Kinetic analyses on both substrates revealed a significantly higher maximum conversion rate for ascorbic acid than for ABTS. The *V*_max_ on ABTS was 11.6 µM/min/mg, while it was 431.19 µM/min/mg on ascorbic acid. Furthermore, the *K*_M_ for ascorbic acid (0.16 mM) was approximately 10-fold lower compared to ABTS (1.9 mM) indicating a higher affinity of *Af_*AO1 for ascorbic acid than for ABTS. Although some laccases have even lower affinity to ABTS than *Af*_AO1, such as laccase from *Klebsiella pneumoniae* with a *K_M_* of 5.33 mM [56], most laccases have a significantly higher specificity for ABTS [57,58]. The affinity of *Af*_AO1 to ascorbic acid is similar to those reported for ascorbate oxidases [51,59]. ASOM, for example, exhibits a *K_M_* of 0.29 mM [15]. The higher specificity and conversion rate for ascorbic acid suggested that *Af_*AO1 is an ascorbate oxidase, but even low activity on ABTS is uncommon for ascorbate oxidases.

Lastly, the three-dimensional structure of *Af_*AO1 was modeled applying a comparative modeling approach using the YASARA modeling suite [60]. The most appropriate templates were identified and include mostly laccase structures (like PDB Codes: 5E9N [38], 5MEW [39], 2QT6 [40], 3DIV [41]), but also an ascorbate oxidase structure was among the best templates (PDB Code: 1ASQ [42]), indicating some sequence similarities of *Af*_AO1 to an ascorbate oxidase. The best template was automatically assigned as the laccase structure from *Steccherinum murashkinskyi* with the PDB-Code 5E9N [38]. Despite the better resolution of this template structure, the second best model using the ascorbate oxidase from *Cucurbita pepo* var. *melopepo* as a template [42], was used as a representative model for *Af*_AO1, because of a better coverage of the alignment (80.7% versus 66.3%) and having a similar overall sequence identity of about 32% (versus 33% to the laccase).

Taken together these results suggest that *Af_*AO1 is an ascorbate oxidase with laccase-like activity. Only very few ascorbate oxidases from fungi have been reported so far, while some untypical ascorbate oxidases from fungi containing heme in the active site are known [18,19,20]. *Af*_AO1 is a copper-containing MCO, exhibiting the conserved copper-coordinating ligands, as well as, 3.3 mol/mol copper. A single copper-containing fungal ascorbate oxidase, ASOM from *Acremonium* sp.-HI-25, is described in literature to date [17]. Furthermore, *Af*_AO1 exhibited an untypical, laccase-like activity, oxidizing ABTS. Similar behavior was only reported for a putative ascorbate oxidase or laccase in *P. anserine* that, if knocked out, decreases the activity of *P. anserine* on ABTS, as well as, on ascorbic acid.

*Af*_AO1 is therefore a two-fold unusual ascorbate oxidase, stemming from fungi and showing activity on ABTS.

## 4. Materials and Methods

### 4.1. Chemicals and Reagents

If not indicated otherwise, chemicals were obtained from Sigma–Aldrich (Munich, Germany).

### 4.2. Expression and Purification of Af_AO1

The gene of *Af*_AO1 was codon optimized for the expression in *Pichia pastoris* and synthesized directly in a pPicZαB vector without its natural signal peptide and without any tag by GenScript (GenScript Biotech, Leiden, The Netherlands). *Af_*AO1 was recombinantly expressed in *P. pastoris* KM71H cells (ThermoFisher Scientific, Waltham, MA, USA). The vector was amplified in *Escherichia coli* BL21-Gold(DE3) (Agilent Technologies, Santa Clara, CA, USA) and isolated with a Promega Midiprep kit (Madison, WI, USA). The vector was linearized with *SacI* (New England Biolabs, Ipswich, MA, USA) and transformed into *P. pastoris* via electroporation (MikroPulserTM, Bio-Rad, Hercules, CA, USA) according to manufacturer’s protocol. Colonies including the insert were determined by colony PCR. Freshly transformed *P. pastoris* cells were cultivated in yeast extract peptone dextrose medium supplemented with 100 µg mL^−1^ zeocin, at 28 °C and 150 rpm, overnight. This culture was used to inoculate 100 mL buffered minimal dextrose medium in 1 L baffled flasks to an OD_600_ of 0.1. The expression was induced after 60 h of incubation at 28 °C with 0.5% methanol twice a day over a period of five days. Additionally, 0.1 mM CuSO_4_ were added with the first induction for the proper folding of the enzyme. The supernatant containing the protein was collected through centrifugation and the protein purified by desalting using an ÄKTApure (GE Healthcare, Little Chalfont, UK) system with a HiPrep™ 26/10 desalting column. A 10 mM sodium acetate buffer pH 5 was applied as buffer. The enzyme concentration was determined photometrically using Bio-Rad Protein Assay (Bio-Rad, Hercules, CA, USA) and bovine serum albumin as protein standard. The purity of the enzyme was confirmed by sodium dodecyl sulfate–polyacrylamide gel electrophoresis (SDS-PAGE) according to Laemmli [61] using precast 4–15% gradient gels (Bio-Rad, Hercules, CA, USA) (Figure S7). As a molecular mass standard pre-stained protein marker IV (Peqlab, Erlangen, Germany) was used. Gels were stained with Coomassie Brilliant Blue.

### 4.3. Characterization of Af_AO1

#### 4.3.1. Sequence Analyses

Protein sequence was on the one hand analyzed manually, by looking for conserved residues, and on the other hand by the conserved domain search of NCBI [37].

#### 4.3.2. Copper Determination

Copper concentration was determined spectrophotometrically (Infinite 200 Pro; Tecan, Männedorf, Switzerland) with a QuantiChrom^TM^ Copper Assay Kit (DICU-250; BioAssay Systems, Hayward, CA, USA) according to manufacturer’s protocol in 96-well plates. Samples had a concentration of 1.1 mg/mL.

#### 4.3.3. Redox Potential

Gold foil surface was activated using 0.1 M KNO_3_ performing 20 cycles from 0.8 to −2.2 V at 0.1 V·s^−1^. A pre-polymerization solution of pyrrole in phosphate-buffered saline (PBS) (5 mL) was prepared by mixing 600 µL enzyme, 194 µL pyrrole and KCl at a final concentration of 0.1 M. The resulting solution was vortexed for 3 min and deoxygenated with nitrogen for at least 30 min, prior to the electrochemical polymerization of pyrrole. The charge accumulated during the electrosynthesis of polypyrrole (PPy) films at 0.85 V was fixed to 100 mC cm^−2^. The gold electrode coated with PPy was cleaned extensively using milliQ water to remove the non-immobilized enzyme and unreacted pyrrole.

PBS was deoxygenated with N_2,_ for at least 30 min, prior to starting the cycling voltammetry experiments in a three-electrode cell using a µAutolab Potentiostat (Ecochemie, Utrecht, The Netherlands). The counter electrode was a platinum wire in spiral geometry and the reference electrode was Ag/AgCl/3 M KCl (Metrohm 6.0726.100). Cyclic voltammograms (CVs) were performed scanning towards positive potentials in a home-made glass cell with a reaction area of 33 mm^2^.

#### 4.3.4. Substrate Screening and Absorption Spectrum

The activity of *Af_*AO1 was determined on 2,6-dimethoxyphenol (DMP; f.c. 0.1 mM), syringaldezine (f.c. 0.05 mM), catechol (f.c. 2 mM), guaiacol (f.c. 20 mM), sinapic acid (f.c. 0.1 mM), ferulic acid (f.c. 0.1 mM), vanillic acid (f.c. 0.1 mM), gallic acid (f.c. 0.1 mM), and tannic acid (f.c. 0.1 mM). Therefore, substrates were solved in ethanol, methanol, water, or buffer, according to their solubility and diluted to their final concentration in buffer. For syringaldezine 100 mM sodium-phosphate buffer pH 6, for catechol 50 mM sodium acetate buffer pH 5 and for all other substrates 100 mM sodium acetate buffer pH 4 was used. Substrates were incubated with 0.1 mg/mL *Af_*AO1. Absorption was determined through a spectrophotometric scan with wavelengths between 200–800 nm in 1 nm steps. Interaction of enzyme and substrate was ascertained by differences in spectra of reactions with enzyme compared to blanks.

For the absorption spectrum *Af*_AO1 was diluted in citrate-phosphate buffer pH 3.4 and measured, as for the substrate screening.

#### 4.3.5. Enzyme Characterization on ABTS

Enzyme activity on ABTS was determined spectrophotometrically, as described previously [62]. The enzyme was mixed with 10 mM ABTS solved in water and the absorbance at 420 nm determined immediately with a plate reader. To determine optima, the enzyme was incubated at temperatures ranging from 20 °C to 60 °C and at a pH range from pH 3 to pH 6. For pH values from pH 3 to 4 citrate-phosphate buffer was used (ε_460nm, pH3–3.5_ = 34.683 L/mmol/cm; ε_460nm, pH3.6–4.5_ = 33.115 L/mmol/cm). Sodium acetate buffer was used for pH values from 4.8 to 5.5 (ε_460nm,pH4.6–5.5_ = 31.332 L/mmol/cm) and sodium phosphate buffer for pH 6 (ε_460nm,pH6_ = 21.103 L/mmol/cm). For the determination of the temperature optimum the pH was set to the optimal pH of 3.4. The absorption was measured in 20 cycles with an interval time of 10 s. One unit (U) of laccase activity was defined as the amount of enzyme producing 1 µmol of reaction product per minute under the conditions specified.

To determine kinetic values enzyme activity was determined at a range of ABTS concentrations from 0.5 mM to 20 mM. The activity measurement was conducted, as described above, at 25 °C. The enzyme was diluted in 0.1 M citrate buffer at pH 3.4. Kinetic values, *V*_max_ and *K*_M_, were determined with a Lineweaver-Burk diagram [63].

#### 4.3.6. Enzyme Characterization on Ascorbic Acid

Activity on ascorbic acid was determined spectrophotometrically (U-2900; Hitachi, Austria) by the decrease of the substrate absorbance, as previously described [64]. Ascorbic acid was diluted to 0.5 M in buffer and incubated for 2 min at 30 °C and 350 rpm and for another 5 min, after the addition of 25 µL *Af_*AO1. The reaction was stopped by the addition of 750 µL HCl at a concentration of 0.2 M and the absorbance was measured at 245 nm (ε_245_ = 10 mL/µmol*cm). For the blank, the enzyme was added together with the HCl after the second incubation. To determine the temperature optimum the temperature of the water bath and photometer were set to 30 °C to 70 °C and the reaction was carried out at the optimal pH of 5. For the different pH values for the pH optimum determination appropriate buffers were chosen. For pH values between 6 and 8 phosphate buffer and for pH values between 3 and 6 sodium acetate buffer was used. To determine enzymatic stability, *Af*_AO1 was incubated at 40 °C and activity measured after 0.5, 1, 2, 4, 5 and 24 h. One unit (U) of ascorbate oxidase activity was defined, identical to one unit of laccase activity.

Kinetic parameters were determined by activity measurements as described, at optimal pH and 30 °C, with ascorbic acid concentrations of 0.05 to 1 mM. With Lineweaver-Burk approximation *V_max_* and *K_M_* were determined [63].

### 4.4. Modeling of Af_AO1

Models of *Af_*AO1 were built based on templates, identified through sequence comparisons. YASARA v.18.2.7.W.64 was used for the building of the models. In total 42 models were built, and the best scoring models were compared manually. The best model was based on the structure of the ascorbate oxidase from *Cucurbita pepo* var. *melopepo* (PDB Code: 1ASQ [42]). This model was chosen because of the overall alignment and the good fit regarding the copper-binding site and the most similar copper and histidine positions in the model compared to the template.

## Figures and Tables

**Table 1:** Substrate screening: *Af*_AO1 converted, of all tested substrates, only ascorbic acid and 2,2′-azino-bis(3-ethylbenzthiazoline-6-sulfonic acid) (ABTS). Activity was measured at conditions preferred by each substrate. Activity was determined at 25 °C, except for ascorbic acid, which was measured at 30 °C.

Substrate	Activity	Buffer
DMP	**-**	sodium acetate pH 4
Syringaldezine	**-**	sodium-phosphate pH 6
Catechol	**-**	sodium acetate pH 5
Guaiacol	**-**	sodium acetate pH 4
Sinapic acid	**-**	sodium acetate pH 4
Ferulic acid	**-**	sodium acetate pH 4
Vanillic acid	**-**	sodium acetate pH 4
Gallic acid	**-**	sodium acetate pH 4
Tannic acid	**-**	sodium acetate pH 4
Ascorbic acid	**+**	sodium acetate pH 4
ABTS	**+**	sodium acetate pH 5

## Supplementary Materials

The following are available online at https://www.mdpi.com/1422-0067/21/16/5754/s1. Figure S1: Wavelength scan of *Af*_AO1 compared to known laccases, Figure S2: Substrate screening, Figure S3: Kinetic parameters of *Af*_AO1, Figure S4: Stability of *Af*_AO1, Figure S5: Alignment *Af*_AO1 with ascorbate oxidase from *Cucurbita pepo* var. *melopepo* (1ASQ), Figure S6: Homology model in cartoon representation compared to the template (PDB-Code 1ASQ), Figure S7: SDS-PAGE of purified and deglycosylated *Af*_AO1.

## Abbreviations

ABTS	2,2′-azino-bis(3-ethylbenzthiazoline-6-sulfonic acid)
*Af*_AO1	MCO from *Aspergillus flavus* (NRRL 3357)
ASOM	ascorbate oxidase from *Acremonium* sp.–HI-25
CV	cyclic voltammogram
DHA	dehydroascorbate
DMP	2,6-dimethoxyphenol
*K_M_*	Michaelis constant
MCO	multicopper oxidase
NHE	normal hydrogen electrode
PBS	phosphate-buffered saline
PPy	polypyrrole
*V_max_*	maximum velocity
