# Supplementary material for: A Fungal Ascorbate Oxidase with Unexpected Laccase Activity
Source: Int J Mol Sci. 2020 Aug 11;21(16):5754. doi: 10.3390/ijms21165754 (PMC7460845; doi:10.3390/ijms21165754)
Supplement: Supplementary file 1 [file ijms-21-05754-s001.pdf]

Supplementary Information

A fungal ascorbate oxidase with unexpected laccase activity

Verena Braunschmid [1,2], Sarah Fuerst [2], Veronika Perz [1], Sabine Zitzenbacher [1], Javier Hoyo [3], Cesar Fernandez-Sanchez [4], Tzanko Tzanov [3], Georg Steinkellner [2,5], Karl Gruber [2,6], Gibson S. Nyanhongo [1,2], Doris Ribitsch [1,2,*], Georg M. Guebitz [1,2]

[1] Institute of Environmental Biotechnology, University of Natural Resources and Life Sciences (BOKU), Tulln an der Donau, Austria
[2] Austrian Centre for Industrial Biotechnology (ACIB), Tulln an der Donau, Austria
[3] Grup de Biotecnologia Molecular i Industrial, Department d'Enginyeria Química, Universitat Politècnica de Catalunya, Rambla Sant Nebridi, España
[4] Instituto de microelectronica de Barcelona (IMB-CNM), CSIC, Campus UAB, Bellatera, Spain
[5] Innophore GmbH, Graz, Austria
[6] Institute of Molecular Bioscience, University of Graz, Austria

* Correspondence: doris.ribitsch@boku.ac.at (D.R.)

Received: date; Accepted: date; Published: date

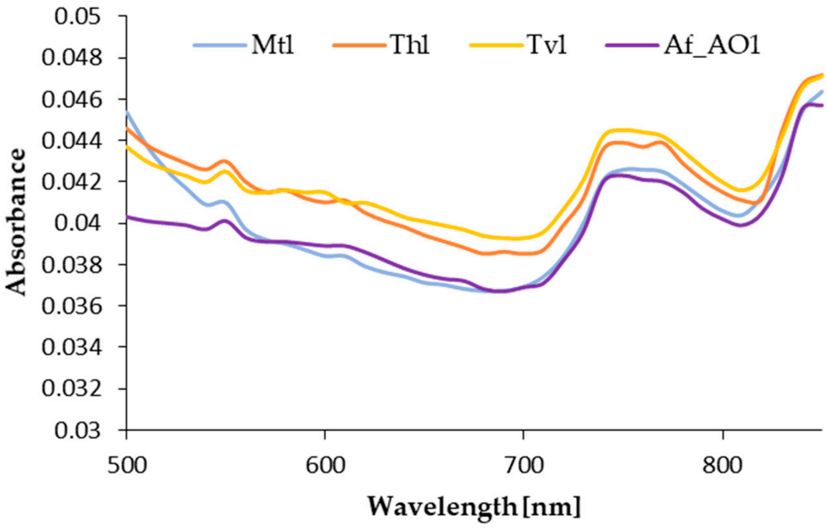

Figure S 1: Wavelength scan of *Af*_AO1 compared to known laccases: Absorbance at wavelength between 500 and 850 nm was measured of *Af*_AO1 and laccases from *Myceliophthora thermophila* (Mtl), *Trametes hirsute* (Thl) and *T. villosa* (Tvl).

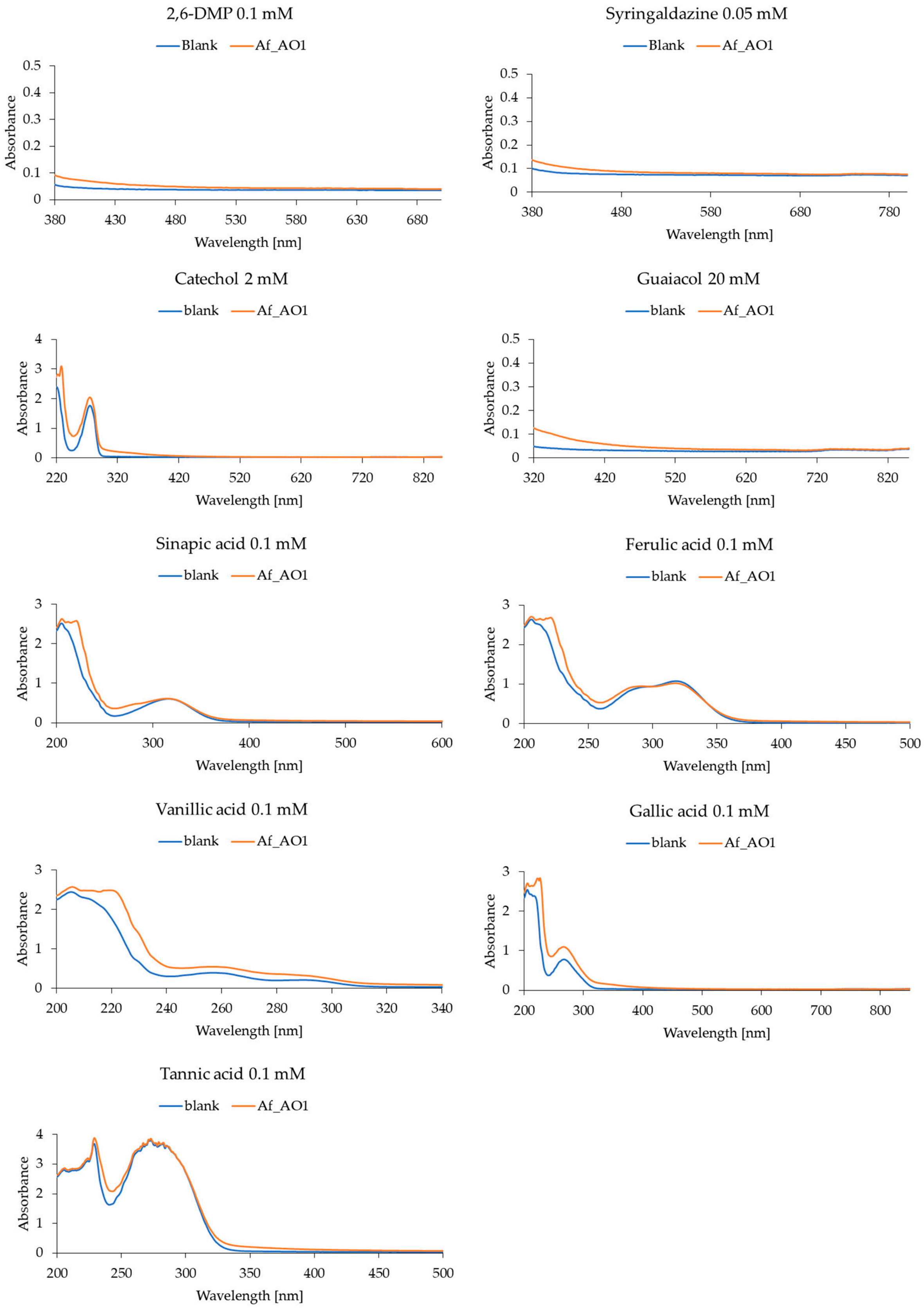

Figure S 2: Substrate screening: Absorption determination of various substrates with and without *Af*_AO1 at wavelength between 200 and 800 nm.

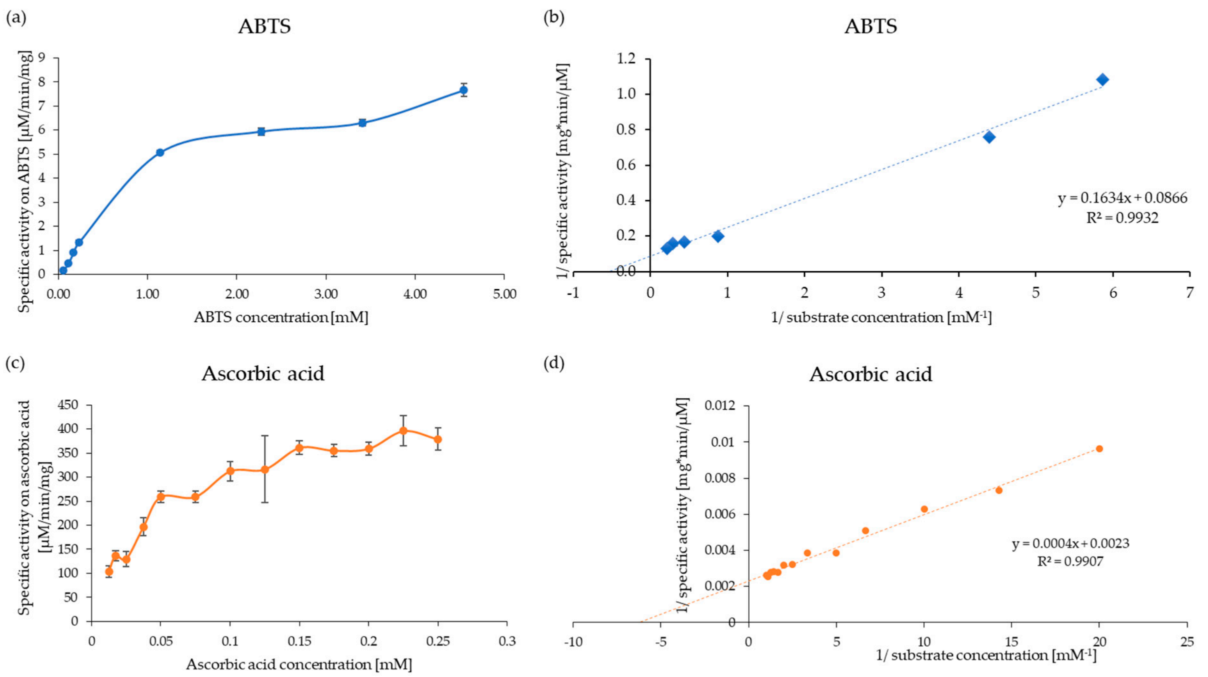

Figure S 3: Kinetic parameters of *Af*_AO1: Kinetic on ABTS as (a) Michaelis-Menten graph and (b) Lineweaver-Burk diagram. Kinetic on ascorbic acid as (c) Michaelis-Menten graph and (d) Lineweaver-Burk diagram. Experiments were conducted in triplicates. The error bars depict standard deviation.

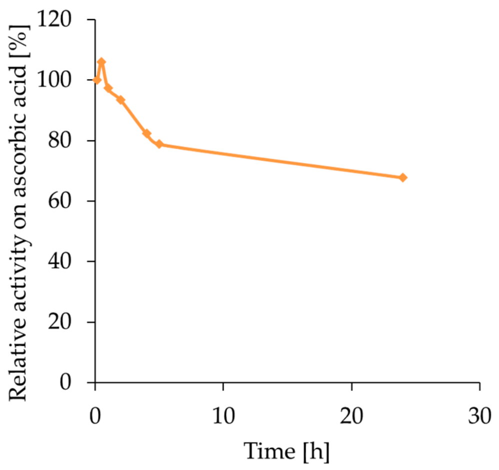

Figure S 4: Stability of *Af*_AO1: Activity on ascorbic acid of *Af*_AO1 during the incubation at 40 °C for 24 h.

```
              ....|....| ....|....| ....|....| ....|....| ....|....|
                      10         20         30         40         50
Af_AO1        HLVLHDDSFQ PDHILRVTAQ DVNQACMDRY SVLINGSLPG PQLNIQEGKV
ASO_CUCPM     -------SQI RHYKWEVEYM FWAPNCNENI VMGINGQFPG PTIRANAGDS
Consensus            S         V         C        ING  PG P      G

              ....|....| ....|....| ....|....| ....|....| ....|....|
                      60         70         80         90        100
Af_AO1        NWIRVYNDME DLNVTMHWHG LSAFTAPFSD GTPMASQWPI PPGHFFDYEV
ASO_CUCPM     VVVELTNKLH TEGVVIHWHG ILQRGTPWAD GTASISQCAI NPGETFFYNF
Consensus           N       V  HWHG       P  D GT   SQ  I  PG  F Y

              ....|....| ....|....| ....|....| ....|....| ....|....|
                     110        120        130        140        150
Af_AO1        RPEVGYAGTY FYHSHVGFQA LT-AWGALIV ESAQ--PSPY QYDEERIIAL
ASO_CUCPM     TVDN--PGTF FYHGHLGMQR SAGLYGSLIV DPPQGKKEPF HYDGEINLLL
Consensus            GT  FYH H G Q       G LIV    Q    P   YD E    L

              ....|....| ....|....| ....|....| ....|....| ....|....|
                     160        170        180        190        200
Af_AO1        SDFFTKTDEE IENGLTSTNF TWSGETSAVL VNGQGRLATN ATGSCKLAAI
ASO_CUCPM     SDWWHQSIHK QEVGLSSKPI RWIGEPQTIL LNGRG----- ----------
Consensus     SD          E GL S     W GE    L  NG G

              ....|....| ....|....| ....|....| ....|....| ....|....|
                     210        220        230        240        250
Af_AO1        SVEPGKTYRL RFIGATALSF VSISLESHDV LEIIEADGHY TKPVNTSYLQ
ASO_CUCPM     ---------- ---------- ---------- ---------- ----------
Consensus

              ....|....| ....|....| ....|....| ....|....| ....|....|
                     260        270        280        290        300
Af_AO1        ISSGQRYSVL LKAKTEAELQ QAKSRQFYFQ LTTMGRPTVL TTFAVLEYPS
ASO_CUCPM     -----QFDCS IAAKYDSNLE PCKLK----- ---------- ----------
Consensus                   AK    L    K

              ....|....| ....|....| ....|....| ....|....| ....|....|
                     310        320        330        340        350
Af_AO1        PTTTDLITVP VTPPLPVANI TYGWLDYTLE PYYPDLDFPT VEEVTRRIII
ASO_CUCPM     ---------- ---------- ---------- ---------- ----------
Consensus

              ....|....| ....|....| ....|....| ....|....| ....|....|
                     360        370        380        390        400
Af_AO1        NVHQNISDRT VWLQNGYDWV ETFPKSPYLV DIYAGTLDLD ASYKRAIASG
ASO_CUCPM     ---------- ---------- ---------- ---------- ----------
Consensus
```

```
....|....| ....|....| ....|....| ....|....| ....|....|
                   410        420        430        440        450
Af_AO1      YAFDNQTRLF PAKMGEVLEI VWQNQGAVSN GGVENHPFHA HGRHFYDIGG
ASO_CUCPM   ---------- ---------- ---------- ---------- ----------
Consensus


            ....|....| ....|....| ....|....| ....|....| ....|....|
                   460        470        480        490        500
Af_AO1      GDGLYNLTEN EARLKGTHPV IRDTTMLYAY RKTTTALEPS GWRAWRIRVT
ASO_CUCPM   ---------- ---------- ---------- ---------- ----------
Consensus


            ....|....| ....|....| ....|....| ....|....| ....|....|
                   510        520        530        540        550
Af_AO1      AAGVWMVHCH VLQHMLMGMQ TAFAFGDQTA IKAQSGTPAE GYLTYGGSAY
ASO_CUCPM   ---------- ---------- ---------- ---------- ----------
Consensus


            ....|....| ....|....| ....|....| ....|....| ....|....|
                   560        570        580        590        600
Af_AO1      GNVTHFPPVK HFFNHLVLHD DSFQPDHILR VTAQDVNQAC MDRYSVLING
ASO_CUCPM   ---------- ---------- ---------- ---------- ----------
Consensus


            ....|....| ....|....| ....|....| ....|....| ....|....|
                   610        620        630        640        650
Af_AO1      SLPGPQLNIQ EGKVNWIRVY NDMEDLNVTM HWHGLSAFTA PFSDGTPMAS
ASO_CUCPM   ---------- ---------- ---------- ---------- ----------
Consensus


            ....|....| ....|....| ....|....| ....|....| ....|....|
                   660        670        680        690        700
Af_AO1      QWPIPPGHFF DYEVRPEVGY AGTYFYHSHV GFQALTAWGA LIVESAQPSP
ASO_CUCPM   ---------- ---------- ---------- ---------- ----------
Consensus


            ....|....| ....|....| ....|....| ....|....| ....|....|
                   710        720        730        740        750
Af_AO1      YQYDEERIIA LSDFFTKTDE EIENGLTSTN FTWSGETSAV LVNGQGRLAT
ASO_CUCPM   ---------- ---------- ---------- ---------- ----------
Consensus


            ....|....| ....|....| ....|....| ....|....| ....|....|
                   760        770        780        790        800
Af_AO1      NATGSCKLAA ISVEPGKTYR LRFIGATALS FVSISLESHD VLEIIEADGH
ASO_CUCPM   -GSESCAPYI FHVSPKKTYR IRIASTTALA ALNFAIG-NH QLLVVEADGN
Consensus       SC       V P KTYR  R    TAL              L   EADG


            ....|....| ....|....| ....|....| ....|....| ....|....|
                   810        820        830        840        850
Af_AO1      YTKPVNTSYL QISSGQRYSV LLKAKTEAEL QQAKSRQFYF QLTTMG-RPT
ASO_CUCPM   YVQPFYTSDI DIYSGESYSV LITTD----- -QNPSENYWV SVGTRARHPN
Consensus   Y  P  TS    I SG  YSV L           Q  S         T    P
```

```
             ....|....| ....|....| ....|....| ....|....| ....|....|
                     860        870        880        890        900
Af_AO1       VLTTFAVLEY PSPTTTDLIT VPVTPPLPVA NITYGWLDYT LEPYYPDLDF
ASO_CUCPM    TPPGLTLLNY LPNSVSKLPT SPPPQTPAWD DFDRSKNFTY RITAAMGSPK
Consensus           L Y        L T  P

             ....|....| ....|....| ....|....| ....|....| ....|....|
                     910        920        930        940        950
Af_AO1       PTVEEVTRRI IINVHQNISD RTVWLQNGYD WVETFPKSPY LVDIYAGTLD
ASO_CUCPM    PPVKFNRRIF LLNTQNVING YVKWAIN--D VSLALPPTPY LGAMKYN---
Consensus    P V    R     N    I      W  N  D      P  PY L

             ....|....| ....|....| ....|....| ....|....| ....|....|
                     960        970        980        990       1000
Af_AO1       LDASYKRAIA SGYAFDNQTR LFPAKMGEVL EIVWQNQGAV SNGGVENHPF
ASO_CUCPM    ---------- ---------- ---------- ---------- ----------
Consensus

             ....|....| ....|....| ....|....| ....|....| ....|....|
                     1010       1020       1030       1040       1050
Af_AO1       HAHGRHFYDI GGGDGLYNLT ENEARLKGTH PVIRDTTMLY AYRKTTTALE
ASO_CUCPM    ---------- ---------- ---------- ---------- ----------
Consensus

             ....|....| ....|....| ....|....| ....|....| ....|....|
                     1060       1070       1080       1090       1100
Af_AO1       PSGWRAWRIR VTAAGVWMVH CHVLQHMLMG MQTAFAFGDQ TAIKAQSGTP
ASO_CUCPM    ---------- ---------- ---------- ---------- ----------
Consensus

             ....|....| ....|....| ....|....| ....|....| ....|....|
                     1110       1120       1130       1140       1150
Af_AO1       AEGYLTYGGS AYGNVTHFPP VKHFFNHLVL HDDSFQPDHI LRVTAQDVNQ
ASO_CUCPM    ---------- ---------- ---------- ---------- ----------
Consensus

             ....|....| ....|....| ....|....| ....|....| ....|....|
                     1160       1170       1180       1190       1200
Af_AO1       ACMDRYSVLI NGSLPGPQLN IQEGKVNWIR VYNDMEDLNV TMHWHGLSAF
ASO_CUCPM    ---------- ---------- ---------- ---------- ----------
Consensus

             ....|....| ....|....| ....|....| ....|....| ....|....|
                     1210       1220       1230       1240       1250
Af_AO1       TAPFSDGTPM ASQWPIPPGH FFDYEVRPEV GYAGTYFYHS HVGFQALTAW
ASO_CUCPM    ---------- ---------- ---------- ---------- ----------
Consensus

             ....|....| ....|....| ....|....| ....|....| ....|....|
                     1260       1270       1280       1290       1300
Af_AO1       GALIVESAQP SPYQYDEERI IALSDFFTKT DEEIENGLTS TNFTWSGETS
ASO_CUCPM    ---------- ---------- ---------- ---------- ----------
Consensus
```

```
             ....|....| ....|....| ....|....| ....|....| ....|....|
                     1310       1320       1330       1340       1350
Af_AO1       AVLVNGQGRL ATNATGSCKL AAISVEPGKT YRLRFIGATA LSFVSISLES
ASO_CUCPM    ---------- ---------- ---------- ---------- ---LLHAFDQ
Consensus

             ....|....| ....|....| ....|....| ....|....| ....|....|
                     1360       1370       1380       1390       1400
Af_AO1       HDVLEIIEAD GHYTKPVNTS YLQISSGQRY SVLLKAKTEA ELQQAKSRQF
ASO_CUCPM    NPPPEVFPED YDIDTPPTNE KTRIGNG--- ---------- ----------
Consensus        E    D      P        I  G

             ....|....| ....|....| ....|....| ....|....| ....|....|
                     1410       1420       1430       1440       1450
Af_AO1       YFQLTTMGRP TVLTTFAVLE YPSPTTTDLI TVPVTPPLPV ANITYGWLDY
ASO_CUCPM    ---------- ---------- ---------- ---------- ----------
Consensus

             ....|....| ....|....| ....|....| ....|....| ....|....|
                     1460       1470       1480       1490       1500
Af_AO1       TLEPYYPDLD FPTVEEVTRR IIINVHQNIS DRTVWLQNGY DWVETFPKSP
ASO_CUCPM    ---------- ---------- ---------- ---------- ----------
Consensus

             ....|....| ....|....| ....|....| ....|....| ....|....|
                     1510       1520       1530       1540       1550
Af_AO1       YLVDIYAGTL DLDASYKRAI ASGYAFDNQT RLFPAKMGEV LEIVWQNQGA
ASO_CUCPM    ---------- ---------- ---------- -VYQFKIGEV VDVILQNANM
Consensus                                          K GEV      QN

             ....|....| ....|....| ....|....| ....|....| ....|....|
                     1560       1570       1580       1590       1600
Af_AO1       VSNGGVENHP FHAHGRHFYD IGGGDGLYNL TENEARLKGT HPVIRDTTML
ASO_CUCPM    MKENLSETHP WHLHGHDFWV LGYGDGKFSA EE-ESSLNLK NPPLRNTVVI
Consensus          E HP  H HG  F    G GDG      E E  L     P  R T

             ....|....| ....|....| ....|....| ....|....| ....|....|
                     1610       1620       1630       1640       1650
Af_AO1       YAYRKTTTAL EPSGWRAWRI RVTAAGVWMV HCHVLQHMLM GMQTAFAFGD
ASO_CUCPM    FPY------- ---GWTAIRF VADNPGVWAF HCHIEPHLHM GMGVVFAEG-
Consensus      Y           GW A R       GVW   HCH   H  M GM   FA G

             ....|....| ....|....| ....|....| ....|..
                     1660       1670       1680
Af_AO1       QTAIKAQSGT PAEGYLTYGG SAYGNVTHFP PVKHFFN
ASO_CUCPM    ----VEKVGR IPTKALACGG TAKSLINNPK NP-----
Consensus            G       L  GG  A
```

Figure S 5: Alignment *Af*_AO1 with ascorbate oxidase from *Cucurbita pepo* var. *melopepo* (1ASQ): Line 1 showes the sequence of *Af*_AO1. Line 2 depicts the sequence of the ascorbate oxidase from *C. pepo* var. *melopepo*, termed ASO CUCPM, aligned to the sequence of *Af*_AO1. The third line exhibits the consensus of the two sequences.

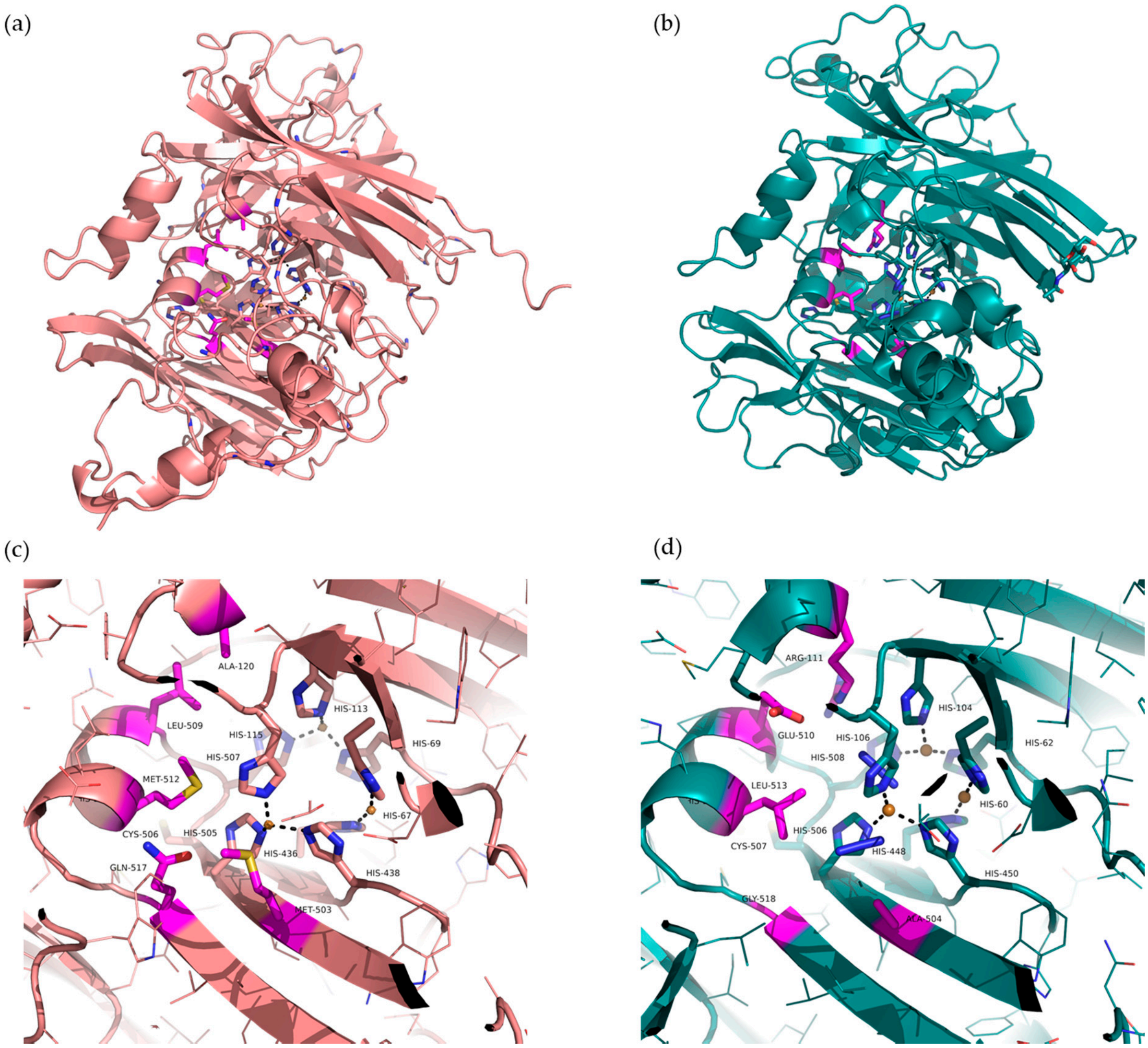

Figure S 6: Homology model in cartoon representation compared to the template (PDB-Code 1ASQ): The amino acid differences near the copper binding site, as well as, the copper binding histidines are labeled or represented as sticks. Amino acid differences are colored in magenta. Overall comparison of (a) the homology model to (b) the template used (PDB-Code 1ASQ). Left: Homology model Right: Template PDB-Code 1ASQ (c) Homology model colored in wheat and zoomed in at the copper binding site. (d) Template with PDB-Code 1ASQ colored in green.

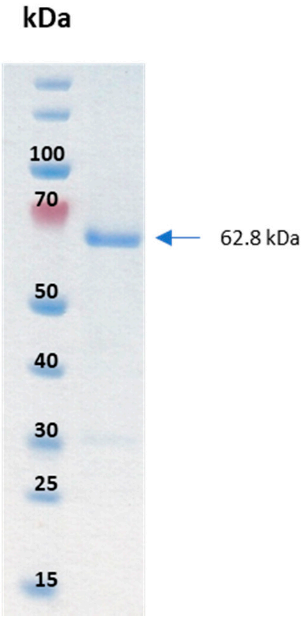

Figure S 7: SDS-PAGE of purified and deglycosylated *Af*_AO1.
